# Re-annotation of 12,495 prokaryotic 16S rRNA 3’ ends and analysis of Shine-Dalgarno and anti-Shine-Dalgarno sequences

**DOI:** 10.1371/journal.pone.0202767

**Published:** 2018-08-23

**Authors:** Mohammad Ruhul Amin, Alisa Yurovsky, Yuping Chen, Steve Skiena, Bruce Futcher

**Affiliations:** 1 Dept. of Computer Science, Stony Brook University, Stony Brook, NY, United States of America; 2 Dept. of Molecular Genetics and Microbiology, Stony Brook University, Stony Brook, NY, United States of America; John Curtin School of Medical Research, AUSTRALIA

## Abstract

We examined 20,648 prokaryotic unique taxids with respect to the annotation of the 3’ end of the 16S rRNA, which contains the anti-Shine-Dalgarno sequence. We used the sequence of highly conserved helix 45 of the 16S rRNA as a guide. By this criterion, 8,153 annotated 3’ ends correctly included the anti-Shine-Dalgarno sequence, but 12,495 were foreshortened or otherwise mis-annotated, missing part or all of the anti-Shine-Dalgarno sequence, which immediately follows helix 45. We re-annotated, giving a total of 20,648 16S rRNA 3’ ends. The vast majority indeed contained a consensus anti-Shine-Dalgarno sequence, embedded in a highly conserved 13 base “tail”. However, 128 exceptional organisms had either a variant anti-Shine-Dalgarno, or no recognizable anti-Shine-Dalgarno, in their 16S rRNA(s). For organisms both with and without an anti-Shine-Dalgarno, we identified the Shine-Dalgarno motifs actually enriched in front of each organism’s open reading frames. This showed to what extent the Shine-Dalgarno motifs correlated with anti-Shine Dalgarno motifs. In general, organisms whose rRNAs lacked a perfect anti-Shine-Dalgarno motif also lacked a recognizable Shine-Dalgarno. For organisms whose 16S rRNAs contained a perfect anti-Shine-Dalgarno motif, a variety of results were obtained. We found one genus, *Alteromonas*, where several taxids apparently maintain two different types of 16S rRNA genes, with different, but conserved, antiSDs. The fact that some organisms do not seem to have or use Shine-Dalgarno motifs supports the idea that prokaryotes have other robust mechanisms for recognizing start codons for translation.

## Introduction

In prokaryotes, ribosomes are assisted in finding a start codon by nucleotide complementarity. About 7 bases upstream of a start codon, there is often a four to six nucleotide “Shine-Dalgarno” (abbreviated here as “SD”) sequence [1–4], recognized by complementary sequences near the 3’ end of the 16S rRNA. For instance, in *E*. *coli*, many open reading frames are preceded by the SD sequence AGGAGG or a subsequence thereof, while the 3’ end of the 16S rRNA has the sequence GATCA**CCUCCU**UA-3’OH (the complement to the Shine-Dalgarno sequence in bold) [2]. We call this complementary sequence in the 16S rRNA the “anti-Shine-Dalgarno” sequence, or “antiSD”. Pairing between the SD sequence on the mRNA, and the antiSD on the 16S rRNA helps position the ribosome to initiate translation [1, 5–8]. This may be especially valuable in poly-cistronic mRNAs [8]. Both the SD sequences and the 16S rRNA sequences are highly conserved [1, 5–8].

While studying SD sequences, we noted that in many prokaryotic genomes, the 3’ ends of 16S rRNAs appeared to be mis-annotated in that the annotated 3’ end occurs only a few nucleotides after the very highly conserved helix 45 [9, 10], just before or inside the antiSD sequence. Similar mis-annotations have been noticed previously [7, 11–13]. However, when we examined the genomic sequence, the full antiSD sequence was often present, and this was also noted previously in 98 cases [7]. We devised a computational pipeline to systematically re-annotate 16S rRNA ends. This pipeline required (a) presence of conserved helix 45; and (b) presence of genomic sequence at least 13 bases 3’ to the end of helix 45. These 13 bases typically include the whole antiSD sequence. In this way, we found a large number (~12,500) of additional prokaryotes with 16S rRNAs containing antiSD sequences. We characterized these, and to some extent the complementary SD sequences. In all but a few rare cases (128), an antiSD sequence existed in the 16S rRNA, and there was usually a corresponding, complementary SD sequence in front of the open reading frames of the organism. However, there were also a significant number of cases where the SD-like sequence in front of genes was shifted one, two or three bases with respect to the16S rRNA. There were also a significant number of cases where no SD-like sequence was found in front of open reading frames. There were 128 cases (out of 20,648) where no antiSD existed in the 16S rRNA(s). These results support suggestions that prokaryotes have other mechanisms for recognizing start codons for translation.

## Results

### Re-annotation increases the number of known anti-Shine-Dalgarno sequences from 8069 to 20,495

As described in Methods, we analyzed ~121,000 prokaryotic 16S rRNA sequences, representing 34,439 unique prokaryotic taxids. Of these, 8,069 taxids were already annotated such that the 16S rRNA contained an antiSD sequence. We were able to re-annotate a further 12,426 taxids, extending the annotation of the 3’ end of the 16S rRNA, so that the newly-annotated 16S rRNA did contain a perfect consensus antiSD, CCUCCU. This increases the number of prokaryotic taxids with antiSD sequences from 8069 to 20,495. There were a further 13,791 taxids that could not be re-annotated (see Methods), and 24 where key sequence information was missing or ambiguous, and finally there were 128 taxids where 16S rRNAs convincingly lacked a consensus antiSD (15 were known previously [14]). 19 of these had a close variant of the consensus antiSD, while 109 had a distant variant or appeared to completely lack an antiSD. These results are summarized in Table 1, and detailed results are given in S1 and S2 Tables, and at our website https://sites.google.com/cs.stonybrook.edu/16s.

**Table 1:** Summary of Re-annotations.

Unique taxids	34,439
No annotated 16S rRNA	11,941
No helix 45 homology in sequence	1,850
Original annotation includes 13 b tail and antiSD	8,080
Annotation corrected by extension through antiSD	12,415
Variant antiSD, missing or ambiguous sequence	25
13 b tail with variant antiSD close to consensus	19
13 b tail, no antiSD-like sequence in tail	109

#### Where is the correct 3’ end?

**Fig 1:**
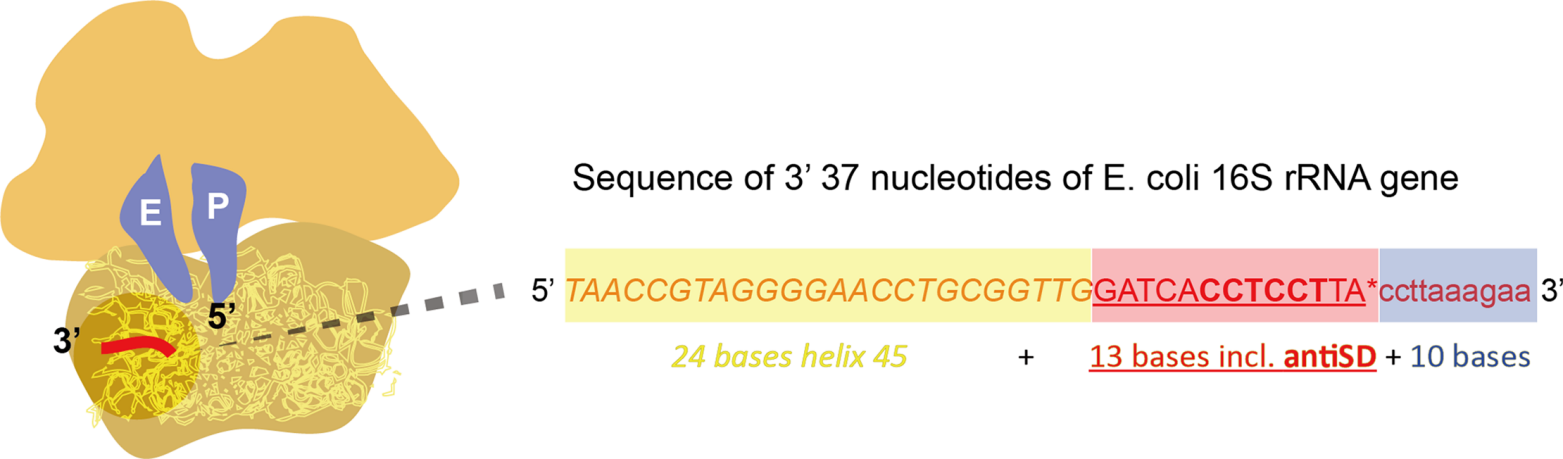
Sequence of 3’ 37 nucleotides of *E*. *coli* 16S rRNA gene. The last 24 nucleotides of helix 45 are in italics (yellow box), followed by the 13 nucleotide “tail” (underlined, red box). The anti-Shine-Dalgarno sequence CCTCCT is underlined and in bold. The terminal 3’ “A” residue is indicated with an asterisk. The 10 genomic nucleotides following the end of the tail are shown in lower case (blue box). The overall positioning of this region of the 16S rRNA is indicated (red line).

In *E*. *coli*, it has been shown that the 3’ end of the 16S rRNA occurs 13 bases after the end of helix 45; the sequence of this 13 base tail is GAUCACCUCCUUA (Fig 1) [2]. However, for most other organisms, the exact 3’ end has not been established. Wei et al. [15] examined 16S rRNA from *B*. *subtilis* by RNA-seq, and found that the tail was GAUCACCUCCUUUCU; that is, a 15 base tail, two bases longer than the *E*. *coli* tail. In our 12,426 re-annotations, we find a perfect consensus CCUCCU antiSD beginning exactly 5 bases after helix 45; we provisionally assume that these 3’ ends are like the 3’ end of E. coli, and end on the 13^th^ base—but we are aware of no evidence for or against the idea that many of these organisms might have 14 or 15 base tails, as in *B*. *subtilis* [15].

In the pre-existing 8069 annotations that do include the antiSD, there is no assumption about the length of the tail after helix 45, and the annotated 3’ ends occur at various apparently arbitrary distances after the end of helix 45.

**Fig 2:**
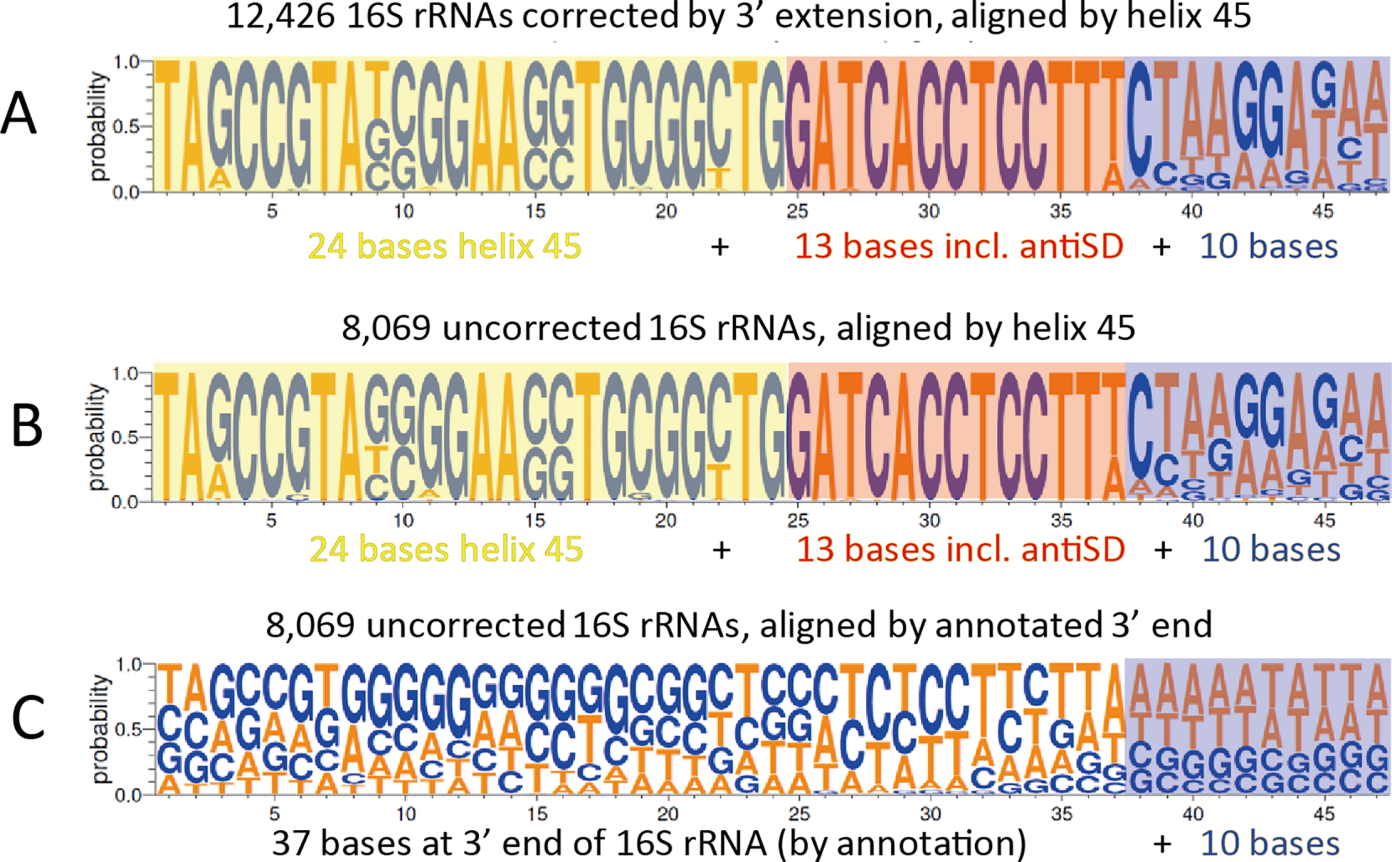
Consensus alignments. In A and B, 24 bases of helix 45 are boxed in yellow; the last 13 bases of the 16S rRNA are boxed in red, and the 10 bases in the genome following the 3’ end of the 16S rRNA are boxed in blue. A. The 12,426 16S rRNAs missing the antiSD as currently annotated and corrected here, aligned by helix 45. B. 8,069 16S rRNAs which include the antiSD as currently annotated, aligned by helix 45. C. The same 8,096 16S rRNAs as in B, but aligned by the 3’ end of the current annotation.

To illustrate the sequence conservation and variation in this tail and adjacent regions, we made three consensus alignments (“Weblogos”) (Fig 2). In the first, Fig 2A, we consider our 12,426 re-annotations. We take the last 24 bases of helix 45, plus the 13 base tail, plus the next 10 bases of the genome, and align by helix 45. The alignment shows striking conservation through the whole 13 base tail, with a small amount of sequence heterogeneity on the last (13^th^) base, with heterogeneity increasing thereafter. In the second consensus alignment, Fig 2B, we repeat the analysis for the existing 8096 annotations, again aligning by helix 45, and get essentially identical results. In the third consensus alignment, Fig 2C, we align the 3’ terminal 47 bases from the 8096 existing annotations, but in this case, we align by the annotated 3’ ends. In this alignment, heterogeneity is seen at every position. Although a rigorous conclusion cannot be reached without experiments, this result suggests that the existing annotations of the 3’ ends are not correct in many cases. That is, in many of these existing annotations, the true 3’ end is likely shorter than annotated.

Because the sequence conservation is very strong up to the 15^th^ base after helix 45 (Fig 2), it seems possible that in various cases, the true 3’ end could plausibly be at least 15 bases from helix 45 [15]. Again, experiments are required to establish this.

The consensus alignments in Fig 2 have limited resolution; minor sequence variants cannot be seen. For our 12,426 re-annotations plus the existing 8096 annotations, we examined the exact sequences of the 13 base tails. Again, it was clear that the 13 base tail is very highly conserved, with only very limited sequence variation. The major variants (> 40 occurrences) and their frequencies are shown in Table 2. Of the 20,495 total sequences, over 19,600 have a tail with one of two sequences: GATCACCTCCTTT (14,088) or GATCACCTCCTTA (5,527). Detailed results are in S2 Table.

**Table 2:** Sequences of 13 base tails.

Tail Sequence	Frequency
GATCACCTCCTTT	14,088
GATCACCTCCTTA	5,527
GAACACCTCCTTT	489
GATTACCTCCTTT	116
GATTACCTCCTAA	87
GAACATCTCATTT	60
GATCACCTCCTAA	53
GAATACCTCCTTT	52
GGTCACCTCCTTT	41

#### What sequence is used as a Shine-Dalgarno?

The SD sequence AGGAGG was originally identified because it is enriched in front of the open reading frames of *E*. *coli*. Similarly, one might expect that AGGAGG or a subsequence might be enriched in front of open reading frames for all the organisms that carry CCUCCU in the tail of the 16S rRNA. However, because these tails have 13 highly-conserved bases (Table 2), it is also possible that the Shine-Dalgarno sequence shifts 5’ or 3’ with respect to this tail. In addition, as noted about, the length of the tail might vary. It is also of interest to ask what, if any, sequences are enriched in front of open reading frames of organisms with a variant or absent antiSD.

Therefore we used the approach of Tompa [16] to ask what sequences are enriched in front of open reading frames of various example prokaryotes. Other methods have been used to find putative SD sequences [1, 17] which depend on the free-energy of hybridization to the known anti-SD sequences, but unlike these, the method of Tompa makes no *a priori* assumptions about what the enriched sequences might be, so is capable of finding variant, shifted, or novel SD motifs, should they exist. We chose 222 examples from diverse phyla for organisms that had a 13 base tail containing CCUCCU. We also examined all 128 organisms that had a variant or absent antiSD. Full results are shown in S3 and S4 Tables, and example results are shown in Tables 3 and 4.

**Table 3:** For CCTCCT antiSDs, examples of Shine-Dalgarno motifs.

Type	Tompa SD	Number	Z-Scores
13 nt a-tail	AAAGGAGGTGATC		
AGGAGG	AGGAGG	14	37–74
	GGAGG	18	7–79
	AGGAG	116	7–71
	AGGA	10	12–25
	GGAG	3	9–39
Shifted	AAGGA	23	13–29
	AAGGAG	11	26–56
	GAGGTG	6	14–58
	GAGGT	6	8–48
	GGTGA	6	21–58
	TGATC	2	11–14
Absent	TACACT	1	43
	TAGACT	1	30
	TATACT	1	43
	CGATCG	3	14–36

SDs are shown in their relative positions along the 13 nucleotide anti-tail (“13 nt a-tail”) (i.e., the strand complementary to the 13 nucleotide tail). SDs are classified into three types: “AGGAGG”, a subset of the classic sequence; “Shifted”, not a subset of the classic sequence, and shifted either 5’ or 3’ along the 13 base tail; and “Absent”, a sequence found by the Tompa algorithm, but not complementary to the tail. The number of each kind of SD (out of 222 examined) found by the Tompa approach is shown (for example, out of 222 species examined by the Tompa method, 14 had the SD sequence AGGAGG). The Z-score is a statistical measure of the significance of the motifs found by the Tompa approach, with larger Z-scores being more significant (Tompa, 1999).

**Table 4:** Presence of Tompa Shine-Dalgarno as function of antiSD.

	Has CCUCCU	%	No CCUCCU	%
SD present	176	79	3	2
SD close	13	6	5	4
SD absent	33	15	120	94
Total	222		128	

222 species that contained a CCUCCU antiSD in their 13 b tails were categorized as to whether the Tompa method found a complementary SD in front of genes, or an almost complementary SD, or no complementary SD at all. 128 species that did not contain a CCUCCU antiSD in their 13 b tails were categorized in the same three ways.

#### Shine-Dalgarno motifs in organisms with a CCUCCU antiSD

First we examined 222 organisms whose tails included CCUCCU (Tables 3 and S3). For 176 (79%), the Tompa algorithm indeed found an upstream motif complementary to the tail. We classify these motifs into two sets. First, for most cases, the Tompa SD motif is complementary to a subset of the CCUCCU antiSD sequence. For example, AGGAGG, AGGA, and GGAG we classify as subsets (Tables 3 and S3). Second, for a significant minority of organisms, the Tompa SD motif is complementary to the 13 base tail, but is shifted 5’ or 3’ with respect to the CCUCCU antiSD. For instance, AAGGA, GGTGA, and TGATC we classify as “shifted” motifs. Note that the shifted motif TGATC does not resemble the classic SD sequence AGGAGG, and does not have complementarity to the classic CCUCCU antiSD, but nevertheless is fully complementary to the tails of the organisms in which it is found, immediately 5’ of the CCUCCU motif. This motif would not be recognized as an SD motif by a method that looked at free energy of hybridization to CCUCCU.

For 46, 21% of the total, the motif found by the Tompa algorithm was not perfectly complementary to any part of the 13 base tail. 13 were very close (within one mismatch) of being complementary, and we classified these as “close”. A further 33 we classified as “absent” (from the tail). Four of these 33 motifs had very high Z-scores (30 or more, Tables 3 and S3), suggesting they may have some function, though they were not complementary to any part of the 3’ end of the 16S rRNA. (They also did not have non-random complementarity to any common other region of the 16S rRNA.) However, most of the 33 motifs had low (poor) Z-scores, suggesting that most likely there was no motif enriched in front of open reading frames in these organisms. This suggests the perhaps surprising conclusion that about a sixth of these 222 organisms, despite having a perfect antiSD in their 16S rRNA, may not use the Shine-Dalgarno mechanism to initiate translation. Other workers have come to similar conclusions [1, 7, 14, 18, 19].

#### Does the length of the tail determine the sequence used as an antiSD?

*E*. *coli* has a 13 b tail after helix 45, and uses an antiSD sequence beginning on the 6^th^ nucleotide after helix 45. Possibly the region of the tail used as an antiSD sequence is a fixed distance from the 3’ end, in which case an organism with a 15 b tail would have a shifted antiSD, UCCUUU, implying an SD sequence of AAAGGA [15, 20]. However, the Tompa method applied to *B*. *subtilis*, which has a 15 b tail [15], nevertheless found the classic SD sequence AGGAGG.

#### Shine-Dalgarno motifs in organisms without a CCUCCU antiSD

We also examined the 128 organisms whose tails did not contain CCUCCU (Tables 4 and S4). Results of the Tompa motif search were quite different from the searches of organisms with CCUCCU, in that now only 3 of the 128 cases (2%) had a Tompa motif that was complementary to any sequence in the 13 base tail, and even then, in these three cases the Tompa motif was the low-complexity, low-affinity sequence AAAA, which we feel is unlikely to function as an SD sequence.

In a further 5 cases, there was a Tompa motif we scored as “close” (1 mismatch) to complementary to the 13 base tail, and these five cases were similar to known SD motifs. In the case of *Mesorhizobium huakuii* 7653R, the 13 base tail was GATCACCTCTTAG (a T-to-C change from consensus antiSD), while the Tompa SD motif was AGGAG with a Z-score of 21.5. In the case of *Streptococcus parasanguinis* the 13 base tail was GATCACCTTCTTT (a C-to-T change from consensus), while the Tompa SD motif was AGGAG with a Z-score of 39.2. Both these could be cases where (a) the antiSD was lost recently in evolution by mutation, and the SD sequence in front of genes still remains; or (b) there is an error in the sequence of the 16S rRNA, and the real sequence has a consensus CCTCCT in the 13 base tail.

The other 120 cases have a Tompa SD motif which is not a close match for anything in the 13 base tail. In these 120 cases, the Z-scores are generally low to moderate, leaving open the possibility that the motif has little functional significance.

The general impression from these 128 organisms is when the classic and exact CCUCCU antiSD is not present in the 13 base tail, then an SD sequence is not present in front of the open reading frames. These are presumably organisms that do not use the Shine-Dalgarno mechanism for initiating translation. We found no cases where a variant antiSD was perfectly complementary to a variant SD (disregarding the three “AAAA” motifs). A similar conclusion was reached by Lim et al. who studied 15 of these organisms lacking a classic antiSD [14].

#### Evolution/Phylogeny

**Fig 3:**
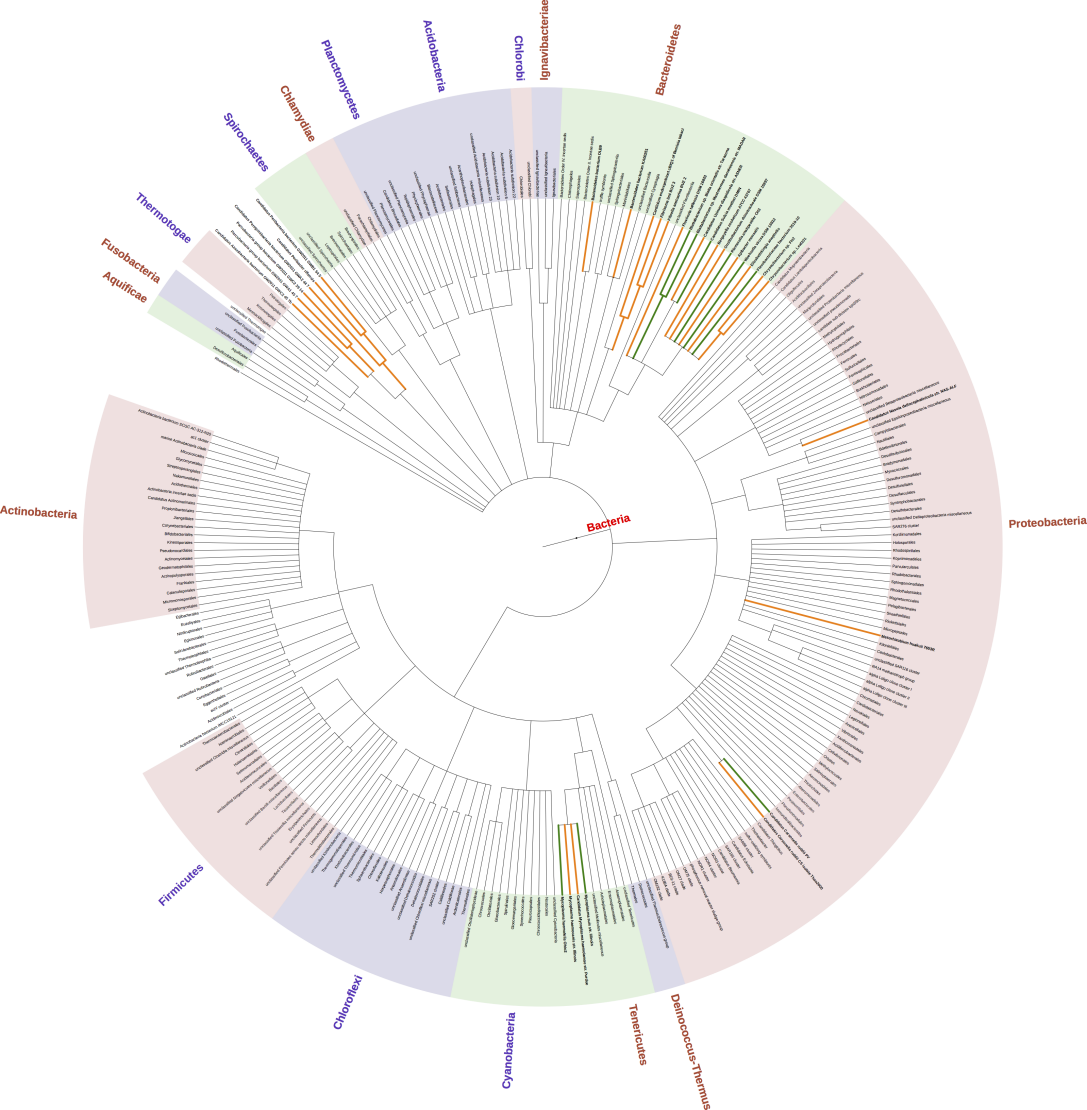
Pylogenetic tree. Phylogenetic tree (low resolution) showing the distribution of the 128 species that lack a CCUCCU antiSD in their 13 b tails. Green lines indicate the phylogenetic positions of the 15 species previously identified by Lim et al. (2012) (and also identified here); orange lines show the other 113 species uniquely identified here. Because of the low resolution of this tree, individual species are not visible (i.e., there are fewer than 128 colored lines).

We examined the phylogenetic distribution of the 128 organisms that lack the CCUCCU antiSD. They were concentrated in the Phylum Bacteroidetes, Genus Chryseobacterium, Elizabethkingia, Riemerella, and Blattabacterium, and also in Tenericutes (Genus Mycoplasma), and Proteobacteria (Genus Candidatus Carsonella), (Fig 3, S1 Fig, S5 Table). Other members of some of these same groups were found as organisms that have a CCUCCU antiSD, but where no matching SD was found by the Tompa method. Thus, these may be groups of organisms that systematically use some alternative method of translation initiation. In addition, sporadic organisms lacking a CCUCCU antiSD, and also lacking a strong SD as defined by the Tompa method, were also found in many other genera (most of these were within the phyla Bacteroidetes, Tenericutes, Firmicutes, or Proteobacteria).

#### Presence of multiple, different tail sequences

In the analysis above, we selected from each taxid the one 16S rRNA sequence that was the best match to the consensus. However, it is possible that some taxids have multiple, different 16S rRNAs. We analyzed 40,235 taxids for the possibility of multiple different 16S rRNA genes. Of these, 14,606 had two or more 16S rRNA genes (with a maximum of twenty-five 16S rRNA genes per genome) (S6 Table).

The number of genes for 16S rRNA did not seem to be characteristic of a species. Very often, the database includes multiple isolates (multiple taxids) with a single species name. Very often, these different isolates of apparently the same species had widely different numbers of 16S rRNA genes (S6 Table). For example, there were 12 taxids with the species name “*Clostridium perfringens*”. Two of these had one 16S rRNA gene; two of them had fifteen 16S rRNA genes; and the other eight had various intermediate numbers, with a mode of 10. As another example, there were about 340 taxids with the species name “*Shigella sonnei*”. About 330 of these had 1 16S rRNA gene, but eight had 4, 6, 7 or 8 16S rRNA genes. It appears that 16S rRNA copy number can vary within different isolates of a single species. However, the apparent number of genes for the 16S rRNA could also vary because of a combination of sequence errors and genome assembly errors.

Of the 14,606 taxids with multiple 16S rRNA genes, 441 (3%) apparently had at least two different sequence classes of 16S rRNA genes (S6 Table). In 100–150 cases, one of the sequence classes was likely due to an error, because in ~65 cases, the sequence contained “N”s and was ambiguous, and in ~65 cases, one sequence was so far from the consensus that we believe it is unlikely to truly represent a 16S rRNA gene. Nevertheless, there were about 300 organisms with two or more plausible different sequence classes of 16S rRNA genes. The majority of these were single base differences, and therefore could simply be sequencing errors. However, in some cases, each of the sequence classes was apparently represented by multiple genes, sometimes with multiple, conserved sequence changes, and this is harder to explain as a sequencing error. Examples of taxids with multiple sequence classes of 16S rRNAs are shown in S7 Table.

We examined a few cases of taxids with multiple different 16S rRNA genes in more detail. For each taxid in S7 Table, we asked whether there were other taxids with the same species name, and if so whether these also had multiple different 16S rRNA genes. In most cases, there were other taxids (but not for *Streptomyces griseorubens*), but in most of these other taxids, there was only one sequence class of 16S rRNA gene.

The striking exception to this was *Alteromonas mediterranea/macleodii*. There were seven taxids of either *Alteromonas mediterranea* or *Alteromonas macleodii* which had multiple (usually two) sequence classes of 16S rRNA genes, and, strikingly, the same sequences were conserved in all seven taxids (Table 5). (In addition, there were other taxids of *Alteromonas* with only one sequence class.) Furthermore, the altered sequence was within the antiSD region. The majority sequence class had the terminal sequence CCUCCUUA (a classic antiSD), while the minority sequence class, conserved in at least two genes in each of seven taxids, had the sequence CCU**U**C**AAU** (sequence changes in bold), with the rest of the 37 base tail perfectly conserved. This suggests that in this organism, there may truly be multiple different conserved 16S rRNA genes, bearing different antiSDs.

**Table 5:** Multiple 16S rRNA genes in *Alteromonas* species.

Species	Taxid	# Genes	Tail Sequence
A. macleodii AD45	GCA_000300175.1	2	TAACCCTAGGGGAACCTGGGGTTGGATCACCTCCTTA
		2	TAACCCTAGGGGAACCTGGGGTTGGATCACCT**T**C**AAT**
		1	TAACCCTAGGGGAACCTGGGGTTGGATCACCT**AAAAT**
A. mediterranea U4	GCA_000439515.1	3	TAACCCTAGGGGAACCTGGGGTTGGATCACCTCCTTA
		2	TAACCCTAGGGGAACCTGGGGTTGGATCACCT**T**C**AAT**
A. mediterranea	GCA_000020585.3	3	TAACCCTAGGGGAACCTGGGGTTGGATCACCTCCTTA
		2	TAACCCTAGGGGAACCTGGGGTTGGATCACCT**T**C**AAT**
A. mediterranea U8	GCA_000439555.1	3	TAACCCTAGGGGAACCTGGGGTTGGATCACCTCCTTA
		2	TAACCCTAGGGGAACCTGGGGTTGGATCACCT**T**C**AAT**
A. macleodii (BS)	GCA_000299995.1	3	TAACCCTAGGGGAACCTGGGGTTGGATCACCTCCTTA
		2	TAACCCTAGGGGAACCTGGGGTTGGATCACCT**T**C**AAT**
A. mediterranea UM4b	GCA_000439595.1	2	TAACCCTAGGGGAACCTGGGGTTGGATCACCTCCTTA
		3	TAACCCTAGGGGAACCTGGGGTTGGATCACCT**T**C**AAT**
A. mediterranea U7	GCA_000439535.1	3	TAACCCTAGGGGAACCTGGGGTTGGATCACCTCCTTA
		2	TAACCCTAGGGGAACCTGGGGTTGGATCACCT**T**C**AAT**
A. mediterranea	GCA_001562295.1	5	TAACCCTAGGGGAACCTGGGGTTGGATCACCTC**A**TTA
A. mediterranea DE1	GCA_000310085.1	5	TAACCCTAGGGGAACCTGGGGTTGGATCACCTCCTTA

Nine taxids from the genus *Alteromonas* are shown. Each taxid represents an independent isolate and sequence. Seven of the taxids have multiple different 16S rRNA genes, differing in the sequence of the tail. Bold residues are residues differing from the majority, classic sequence. Other taxids of *Alteromonas* (not shown) (S6 Table) have the same tail sequence as *A*. *mediterranea DE1* (GCA_000310085.1), which we refer to as the “classic” or “majority” sequence, and which contains the classic antiSD sequence CCTCCT. There is no taxid of *Alteromonas* in our dataset that contains only the novel tail sequence CCTTCAAT; rather, this novel tail is found only in conjunction with the classic sequence.

We therefore used the Tompa algorithm to characterize the SD sequences in species of *Alteromonas* with two sequence classes of 16S rRNA tail, and in species with just one sequence class. The Tompa algorithm found essentially identical SD motifs in both kinds of species; that is, there is no evidence that any SD sequences in the species with two sequence classes of 16S rRNA have drifted to match the second, novel sequence.

The consensus SD motif found by the Tompa algorithm was AAGGAGA. The best match to the majority (classic) antiSD is therefore:

5’ TCACCTCCTTA 3’

3 aGAGGAA 5’

A mismatch is indicated by a lower case letter. Interestingly, the best match to the minority (novel, changes in bold) antiSD is likewise 6 of 7, but, strikingly, is in a different register:

5’ TCACCT**T**C**AAT** 3’

3’ AGaGGAA 5’

AAGGAGA is a consensus SD sequence. The Tompa algorithm also outputs 20 submotifs on which the consensus is based. If the novel antiSD were ever to be perfectly matched to an SD, that SD would have the sequence AAGGTGA. However, no such submotif was found by the Tompa algorithm for any of the seven taxids carrying the novel antiSD. Furthermore, we searched for *Alteromonas* genes with the motif AGGTG 6, 7, or 8 bases in front of annotated start codons. In *Alteromonas* species with the novel 16S rRNA, there were a mean of 34 such genes; in the *Alteromonas* species without, there were a mean of 31 such genes (not significantly different). The ~30 genes with the AGGTG motif could be specific for the novel antiSD, but then it is hard to understand why their frequency is the same in species with and without the novel antiSD. On the other hand, the SD submotif AAGGAGG was common in all *Alteromonas* species; this submotif is a perfect 7/7 match to the majority (classic) antiSD, but is a relatively poor (5/7) match to the novel antiSD.

In summary, at least seven taxids of *Alteromonas* species appear to carry two different sequence classes of 16S rRNA genes, with different antiSD sequences. Both types of antiSD sequences could bind similarly to most SDs in *Alteromonas*, but in different registers. Some *Alteromonas* genes may be specific for the classic antiSD, and so be specific for one kind of 16S rRNA. The existence of the novel 16S rRNA in at least 7 taxids argues for some functional significance.

One other interesting example of a species with multiple 16S rRNA genes was *Candidatus Hodgkinia cicadicola*, where there was one taxid (of three) with seven 16S rRNA genes, falling into six different sequence classes (S7 Table). The sequence changes occurred mostly in the last 10 bases of the tail, the region that usually includes the antiSD motif. However, this species does not appear to use the Shine-Dalgarno mechanism (see above), and none of these 16S rRNAs include a classic antiSD (see above). The frequent sequence changes in this region could mean that in this organism, this region of the gene is not represented in the mature 16S rRNA, and thus is relatively free to change.

We also used the Tompa algorithm to characterize SD sequences for other species of interest in S7 Table (*Shigella sonnei*, *Clostridiium perfringens*, *S*. *griseorubens*, *M*. *luteus*, *B*. *subtilis*, *M*. *bacterium BACL14*), but in no case did we find a novel SD motif perfectly matching the novel antiSD.

#### Effect of sequencing errors

Since we have considered a large amount of sequence data, and found some organisms with rare properties, we have considered what effect sequencing errors might have on our findings. Indeed, some of the organisms apparently lacking the CCUCCU antiSD might be erroneously categorized in that way due to sequencing errors, and *Mesorhizobium huakuii* 7653R and *Streptococcus parasanguinis*, considered above, could be examples. However, we believe the overall impact of such errors is small, for four reasons. First, many organisms have multiple 16S rRNA genes, and we consider the gene most similar to the CCUCCU consensus. Mis-categorization would require identical sequencing errors in each gene. Second, some of the organisms have been sequenced multiple times. Again, mis-categorization would require multiple identical sequencing errors. Third, if the lack of a CCUCCU antiSD were often a sequencing error, then in most such cases, a consensus SD would still be found for that organism by the Tompa method, but this is not the case (S4 Table). Instead, in almost all cases where a classic antiSD is absent, Shine-Dalgarnos (as found by the Tompa method) are also absent. Fourth, and most persuasively, the organisms lacking the CCUCCU antiSD typically fall into closely-related families (see above). If sequencing errors were mainly responsible for the lack of a CCUCCU antiSD, then organisms categorized this way should fall randomly into the phylogenetic tree, which clearly they do not (Fig 3, S1 Fig, S5 Table).

## Discussion

We have re-annotated 12,495 16S rRNA 3’ ends, increasing the total number of prokaryotes with 16S rRNAs containing antiSD sequences from 8,153 to 20,648, and increasing the number of organisms known to lack an antiSD from 15 [14] to 128. We also used a consensus-motif approach to find the SD sequences actually enriched in front of open reading frames of several hundred example organisms. The vast majority of organisms contain a consensus CCUCCU antiSD embedded in a highly conserved 13 base 3’ tail, and the majority of organisms also contain consensus SD motifs in front of their genes. However there are a significant number of striking exceptions. First, there are a significant number of organisms where the enriched sequence found in front of genes is not the classic AGGAGG, but instead is “shifted” 5’ or 3’ with respect to the CCUCCU antiSD. These organisms are presumably using the Shine-Dalgarno mechanism, but with variant Shine-Dalgarno/anti-Shine-Dalgarno sequences.

Second, we found one genus, *Alteromonas*, in which some taxids may maintain two different sequence classes of 16S rRNA, bearing different antiSD motifs. Possibly, though as yet there is no evidence for this, different 16S rRNAs could be somewhat specific for different SDs of different genes.

Third, for the organisms that do contain a consensus CCUCCU antiSD, about one-sixth do not seem to have any complementary SD sequence enriched in front of their open reading frames. This suggests that perhaps as many as a sixth of organisms do not use the Shine-Dalgarno mechanism to initiate translation for most of their genes. It is already known that even amongst organisms that do use the Shine-Dalgarno mechanism, many individual genes are not preceded by an SD-like motif. Previous workers have also discussed this issue [1, 7, 14, 18, 19].

Fourth, and reinforcing the point above, we found 128 organisms that seem to entirely lack the 16S rRNA antiSD sequence, and which do not have significant motifs in front of open reading frames complementary to any part of the 13 base 16S rRNA tail. Although a few of these organisms may be categorized in this way due to sequencing error, it appears that the majority are not using the Shine-Dalgarno mechanism at all.

What, then, are other mechanisms that could be used for identifying initiation AUGs? One mechanism involves interactions between the 5’ UTR and rribosomal protein S1 [7, 21–23]. Another is a distinct mechanism for translation of leaderless mRNAs [7, 24, 25]. However, the most general idea is that there is little mRNA secondary structure near start AUGs (allowing the ribosome to find such AUGs), while there is more secondary structure in the body of the mRNA and near other, non-start AUGs (and so shields the ribosomes from finding these AUGs [6, 18, 26–31]. Overall, our results emphasize the importance and generality of these non-Shine-Dalgarno mechanisms.

## Materials and methods

### Method for correcting 16S rRNA sequence annotation

We used a simple approach to analyze the 16S rRNA from bacterial genome sequences downloaded in the form of Generic Feature Format (GFF) and FASTA file for DNA Sequences (FNA) from NCBI Genbank. There were a total of 70,473 prokaryotic genomes in the repository at the time of analysis. We used the annotation information from the GFF file to extract the 16S rRNA from the FNA file. This yielded ~121,000 16S rRNA sequences, since many organisms had more than one 16S rRNA gene. There were about 27,428 genomes for which no 16S rRNA was reported in the GFF file. The ETE toolkit was used to get the phylogeny of the organisms. To examine and correct the annotations of the sequences, we used the location of the very highly conserved helix 45 of 16S rRNA. Helix 45 occurs near the 3’ end of the 16S rRNA, and in *E*. *coli* there are 13 nucleotides after helix 45 before the 3’ terminus [2, 9, 10, 15].

For each 16S rRNA annotation in the GFF file of a bacterial genome, we extracted a sequence extended by 500 bases 5’ and 3’ to the annotated 16S rRNA from the respective FNA file. We searched for alignments of helix 45 considering a base matching score of 1, base complement matching score of 0.25 (‘A’ to ‘T’, ‘C’ to ‘G’ and vice versa) and base mismatch score of 0. For all the alignment locations that resulted in a minimum homology score greater than or equal to 18.0, we searched for an anti-Shine-Dalgarno motif starting five bases 3’ to helix 45. We recorded the number of base matches to the anti-Shine-Dalgarno. We also recorded the location of helix 45, and the sequence of the 23 bases 3’ to helix 45 (i.e., including 10 bases that would normally be beyond the 3’ end of the 16S rRNA) for further analysis (Fig 1).

For organisms with multiple 16S rRNA genes, we chose one gene with the highest homology score to helix 45 plus the Shine-Dalgarno sequence (usually, all copies of a gene had the same sequence). This resulted in 34,439 16S rRNA records, one per unique taxid. We found that a total of 8,069 taxids were annotated so as to contain the antiSD. In these 8,069 bacteria, as annotated, we found a helix 45 followed by at least 13 bases, which included the exact classic anti-Shine-Dalgarno motif, CCUCCU. For 12,426 prokaryotes we found helix 45, but the annotated 3’ end of the 16S rRNA was apparently too short, generally occurring about 8 bases 3’ from the end of helix 45, and cutting off part of an anti-Shine Dalgarno sequence. We corrected the annotations for these 12,426 prokaryotes by extending the original annotation, usually by five nucleotides (S1 Table), thus generating a corrected annotation with a helix 45 followed by 13 bases usually including a perfect or very high quality anti-Shine-Dalgarno motif. There were 1,850 bacteria where we did not find helix 45 in the annotated 16S rRNA; we do not know whether these 16S rRNAs genuinely lack helix 45, or whether this is a problem of sequencing or annotation. There were a total of 11,941 taxids for which we did not find any annotated 16S rRNA. Finally, there were 153 bacterial genomes where we found a 16S rRNA with an excellent match to helix 45, but no match (134 organisms) or a non-consensus match (19 organisms) to the anti-Shine-Dalgarno sequence. These were inspected manually. In one case (*Enterococcus faecium* UC7256) a perfect antiSD was found at a variant position, beginning 4 bases from the end of helix 45 instead of 5 bases. In the other 152 cases, no better anti-Shine-Dalgarno-like sequences were seen. 25 of these 153 variant 16S rRNA sequences had missing or ambiguous sequence data, leaving 128 organisms with variant or absent anti-Shine-Dalgarnos. These re-annotations are summarized in Table 1.

Relevant sequence, source, re-annotation details, and other detailed data for these 34,439 16S rRNAs (one per taxid) are given in S2 Table.

### Shine-Dalgarno identification

The putative Shine-Dalgarno (SD) sequences for each bacterium were discovered using the algorithm of M. Tompa [16]. We acquired the program source code from the author and followed the attached instructions. We pre-processed the data to collect short DNA sequences upstream of each gene. We then ran RBSidentify, considering patterns of length 7, and allowing for 1 mis-match. This program outputs the z-scores listing the probability of each 7-mer to occur randomly in the given list of upstream sequences. We only consider the bacteria where the top 20 7-mers have a z-score greater than 5. We then ran RBSprofile on the top 20 7-mers to identify the most significant positions in the unified motif, measured by the relative entropy, reading off the putative SD sequences from the generated matrix.

We then asked whether the complement (the antiSD) of the sequence identified by the Tompa algorithm exists near the 3' end of the corrected 16S rRNA sequence. The result is entered as “yes” if true, and “no” if false, of the “found in 16S” column of tables of results.

## Supporting information

S1 Fig Phylogenetic tree (higher resolution) showing the distribution of the 128 species that lack a CCUCCU antiSD in their 13 b tails. Green lines show the 15 species previously identified by Lim et al. (2012) (and also identified here); orange lines show the other 113 species uniquely identified here.
(PDF)

S1 Table For each of the 12,426 re-annotations, the number of bases by which the previously-annotated tail was extended. For example, there were 10,711 re-annotations where the 3’ end of the tail was extended by 5 bases, and 14 re-annotations where the 3’ end of the tails was extended by 7 bases.
(DOCX)

S2 Table Detailed results. Self-explanatory.
(XLSX)

S3 Table “SD (Tompa)” is the consensus SD motif as found by the Tompa algorithm. “Tompa Z” is the Z-score (for significance) of that motif; a higher score is more significant. “found in 16S” is whether (yes or no or “close”) the SD motif found in the “SD (Tompa)” column is found (antisense) in the 13 base 16S tail of the organism in question.
(XLSX)

S4 Table A list of organisms where an antiSD is not found in the 13 base 16S tail. “SD (Tompa)” and “Tompa Z” and “found in 16S” are as in S3 Table.
(XLSX)

S5 Table Self-Explanatory. (XLSX)

S6 Table Organisms with multiple different 16S tails. “16S count” is the number of annotated 16S rRNA genes. “Number of different ends” is the number of different ends, by sequence, amongst the 16S rRNA genes. “Max Number of Similar Ends” is the maximum number of 16S rRNA genes with the same tail sequence, and the sequence of this most common tail is given in the next column, “Most Similar Sequence”. Subsequent columns contain the sequences of the less-frequent tails, and the count of each of those sequence types is given. For example, for the top entry, *C*. *hodgkinia*, there are 7 annotated 16S rRNA genes, and these have a total of 6 different tails. Two of the genes have sequence TAGCCGTAGGTGAACCTGTGGCTGAAACATAACAACC, while the other five genes each have a unique sequence.
(XLSX)

S7 Table Example taxids from S6 Table. (XLSX)

## References

[1] Ma J, Campbell A, Karlin S. Correlations between Shine-Dalgarno sequences and gene features such as predicted expression levels and operon structures. J Bacteriol. 2002;184(20):5733–45. 10.1128/JB.184.20.5733-5745.2002 ; PubMed Central PMCID: PMCPMC139613. 12270832 PMC139613

[2] Shine J, Dalgarno L. The 3'-terminal sequence of Escherichia coli 16S ribosomal RNA: complementarity to nonsense triplets and ribosome binding sites. Proc Natl Acad Sci U S A. 1974;71(4):1342–6. ; PubMed Central PMCID: PMCPMC388224. 4598299 10.1073/pnas.71.4.1342 PMC388224

[3] Shine J, Dalgarno L. Terminal-sequence analysis of bacterial ribosomal RNA. Correlation between the 3'-terminal-polypyrimidine sequence of 16-S RNA and translational specificity of the ribosome. Eur J Biochem. 1975;57(1):221–30. . 809282 10.1111/j.1432-1033.1975.tb02294.x

[4] Shine J, Dalgarno L. Determinant of cistron specificity in bacterial ribosomes. Nature. 1975;254(5495):34–8. . 803646 10.1038/254034a0

[5] Hockenberry AJ, Pah AR, Jewett MC, Amaral LA. Leveraging genome-wide datasets to quantify the functional role of the anti-Shine-Dalgarno sequence in regulating translation efficiency. Open Biol. 2017;7(1). 10.1098/rsob.160239 ; PubMed Central PMCID: PMCPMC5303271. 28100663 PMC5303271

[6] Li GW. How do bacteria tune translation efficiency? Curr Opin Microbiol. 2015;24:66–71. 10.1016/j.mib.2015.01.001 ; PubMed Central PMCID: PMCPMC4678177. 25636133 PMC4678177

[7] Nakagawa S, Niimura Y, Miura K, Gojobori T. Dynamic evolution of translation initiation mechanisms in prokaryotes. Proc Natl Acad Sci U S A. 2010;107(14):6382–7. 10.1073/pnas.1002036107 ; PubMed Central PMCID: PMCPMC2851962. 20308567 PMC2851962

[8] Osterman IA, Evfratov SA, Sergiev PV, Dontsova OA. Comparison of mRNA features affecting translation initiation and reinitiation. Nucleic Acids Res. 2013;41(1):474–86. 10.1093/nar/gks989 ; PubMed Central PMCID: PMCPMC3592434. 23093605 PMC3592434

[9] Van Knippenberg PH, Van Kimmenade JM, Heus HA. Phylogeny of the conserved 3' terminal structure of the RNA of small ribosomal subunits. Nucleic Acids Res. 1984;12(6):2595–604. ; PubMed Central PMCID: PMCPMC318692. 6709501 10.1093/nar/12.6.2595 PMC318692

[10] Tu C, Zhou X, Tarasov SG, Tropea JE, Austin BP, Waugh DS, et al The Era GTPase recognizes the GAUCACCUCC sequence and binds helix 45 near the 3' end of 16S rRNA. Proc Natl Acad Sci U S A. 2011;108(25):10156–61. 10.1073/pnas.1017679108 ; PubMed Central PMCID: PMCPMC3121871. 21646538 PMC3121871

[11] Lin YH, Chang BC, Chiang PW, Tang SL. Questionable 16S ribosomal RNA gene annotations are frequent in completed microbial genomes. Gene. 2008;416(1–2):44–7. 10.1016/j.gene.2008.02.023 . 18420359

[12] Jones CE, Brown AL, Baumann U. Estimating the annotation error rate of curated GO database sequence annotations. BMC Bioinformatics. 2007;8:170 10.1186/1471-2105-8-170 ; PubMed Central PMCID: PMCPMC1892569. 17519041 PMC1892569

[13] Lagesen K, Hallin P, Rodland EA, Staerfeldt HH, Rognes T, Ussery DW. RNAmmer: consistent and rapid annotation of ribosomal RNA genes. Nucleic Acids Res. 2007;35(9):3100–8. 10.1093/nar/gkm160 ; PubMed Central PMCID: PMCPMC1888812. 17452365 PMC1888812

[14] Lim K, Furuta Y, Kobayashi I. Large variations in bacterial ribosomal RNA genes. Mol Biol Evol. 2012;29(10):2937–48. 10.1093/molbev/mss101 ; PubMed Central PMCID: PMCPMC3457768. 22446745 PMC3457768

[15] Wei Y, Silke JR, Xia X. Elucidating the 16S rRNA 3' boundaries and defining optimal SD/aSD pairing in Escherichia coli and Bacillus subtilis using RNA-Seq data. Sci Rep. 2017;7(1):17639 10.1038/s41598-017-17918-6 ; PubMed Central PMCID: PMCPMC5732282. 29247194 PMC5732282

[16] Tompa M. An exact method for finding short motifs in sequences, with application to the ribosome binding site problem. Proc Int Conf Intell Syst Mol Biol. 1999:262–71. . 10786309

[17] Osada Y, Saito R, Tomita M. Analysis of base-pairing potentials between 16S rRNA and 5' UTR for translation initiation in various prokaryotes. Bioinformatics. 1999;15(7–8):578–81. . 10487865 10.1093/bioinformatics/15.7.578

[18] Burkhardt DH, Rouskin S, Zhang Y, Li GW, Weissman JS, Gross CA. Operon mRNAs are organized into ORF-centric structures that predict translation efficiency. Elife. 2017;6 10.7554/eLife.22037 ; PubMed Central PMCID: PMCPMC5318159. 28139975 PMC5318159

[19] Chang B, Halgamuge S, Tang SL. Analysis of SD sequences in completed microbial genomes: non-SD-led genes are as common as SD-led genes. Gene. 2006;373:90–9. 10.1016/j.gene.2006.01.033 . 16574344

[20] Abolbaghaei A, Silke JR, Xia X. How Changes in Anti-SD Sequences Would Affect SD Sequences in Escherichia coli and Bacillus subtilis. G3 (Bethesda). 2017;7(5):1607–15. 10.1534/g3.117.039305 ; PubMed Central PMCID: PMCPMC5427494. 28364038 PMC5427494

[21] Boni IV, Isaeva DM, Musychenko ML, Tzareva NV. Ribosome-messenger recognition: mRNA target sites for ribosomal protein S1. Nucleic Acids Res. 1991;19(1):155–62. ; PubMed Central PMCID: PMCPMC333546. 2011495 10.1093/nar/19.1.155 PMC333546

[22] Komarova AV, Tchufistova LS, Dreyfus M, Boni IV. AU-rich sequences within 5' untranslated leaders enhance translation and stabilize mRNA in Escherichia coli. J Bacteriol. 2005;187(4):1344–9. 10.1128/JB.187.4.1344-1349.2005 ; PubMed Central PMCID: PMCPMC545611. 15687198 PMC545611

[23] Salah P, Bisaglia M, Aliprandi P, Uzan M, Sizun C, Bontems F. Probing the relationship between Gram-negative and Gram-positive S1 proteins by sequence analysis. Nucleic Acids Res. 2009;37(16):5578–88. 10.1093/nar/gkp547 ; PubMed Central PMCID: PMCPMC2760812. 19605565 PMC2760812

[24] Grill S, Moll I, Giuliodori AM, Gualerzi CO, Blasi U. Temperature-dependent translation of leaderless and canonical mRNAs in Escherichia coli. FEMS Microbiol Lett. 2002;211(2):161–7. . 12076807 10.1111/j.1574-6968.2002.tb11219.x

[25] Moll I, Grill S, Gualerzi CO, Blasi U. Leaderless mRNAs in bacteria: surprises in ribosomal recruitment and translational control. Mol Microbiol. 2002;43(1):239–46. . 11849551 10.1046/j.1365-2958.2002.02739.x

[26] de Smit MH, van Duin J. Secondary structure of the ribosome binding site determines translational efficiency: a quantitative analysis. Proc Natl Acad Sci U S A. 1990;87(19):7668–72. ; PubMed Central PMCID: PMCPMC54809. 2217199 10.1073/pnas.87.19.7668 PMC54809

[27] de Smit MH, van Duin J. Translational initiation on structured messengers. Another role for the Shine-Dalgarno interaction. J Mol Biol. 1994;235(1):173–84. . 8289239 10.1016/s0022-2836(05)80024-5

[28] Del Campo C, Bartholomaus A, Fedyunin I, Ignatova Z. Secondary Structure across the Bacterial Transcriptome Reveals Versatile Roles in mRNA Regulation and Function. PLoS Genet. 2015;11(10):e1005613 10.1371/journal.pgen.1005613 ; PubMed Central PMCID: PMCPMC4619774. 26495981 PMC4619774

[29] Hall MN, Gabay J, Debarbouille M, Schwartz M. A role for mRNA secondary structure in the control of translation initiation. Nature. 1982;295(5850):616–8. . 6799842 10.1038/295616a0

[30] Scharff LB, Childs L, Walther D, Bock R. Local absence of secondary structure permits translation of mRNAs that lack ribosome-binding sites. PLoS Genet. 2011;7(6):e1002155 10.1371/journal.pgen.1002155 ; PubMed Central PMCID: PMCPMC3121790. 21731509 PMC3121790

[31] Wikstrom PM, Lind LK, Berg DE, Bjork GR. Importance of mRNA folding and start codon accessibility in the expression of genes in a ribosomal protein operon of Escherichia coli. J Mol Biol. 1992;224(4):949–66. . 1569581 10.1016/0022-2836(92)90462-s

