# Supplementary figures and images for: Re-annotation of 12,495 prokaryotic 16S rRNA 3’ ends and analysis of Shine-Dalgarno and anti-Shine-Dalgarno sequences
Source: PLoS One. 2018 Aug 23;13(8):e0202767. doi: 10.1371/journal.pone.0202767 (PMC6107228; doi:10.1371/journal.pone.0202767)

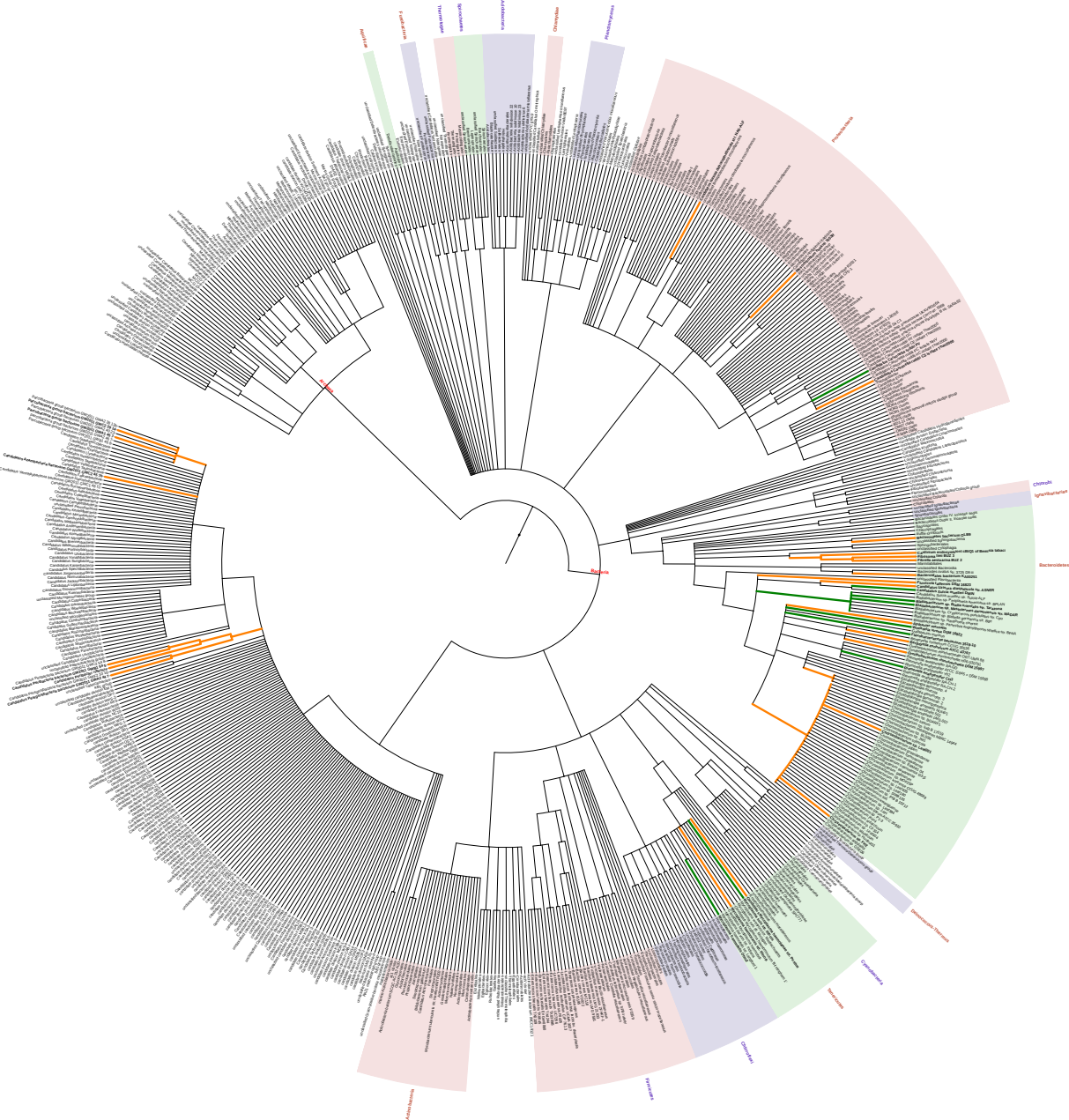

Supplement: S1 Fig — Green lines show the 15 species previously identified by Lim et al. (2012) (and also identified here); orange lines show the other 113 species uniquely identified here. (PDF) [file pone.0202767.s001.pdf]
