# Supplementary material for: Re-annotation of 12,495 prokaryotic 16S rRNA 3’ ends and analysis of Shine-Dalgarno and anti-Shine-Dalgarno sequences
Source: PLoS One. 2018 Aug 23;13(8):e0202767. doi: 10.1371/journal.pone.0202767 (PMC6107228; doi:10.1371/journal.pone.0202767)
Supplement: S1 Table — For example, there were 10,711 re-annotations where the 3’ end of the tail was extended by 5 bases, and 14 re-annotations where the 3’ end of the tails was extended by 7 bases. (DOCX) [file pone.0202767.s002.docx]

Table S1. Lengths of extensions of annotations

| Extension (bases) | Count |
| --- | --- |
| 1 | 372 |
| 2 | 67 |
| 3 | 114 |
| 4 | 31 |
| 5 | 10711 |
| 6 | 391 |
| 7 | 14 |
| 8 | 2 |
| 9 | 2 |
| 10 | 6 |
| 11 | 2 |
| 12 | 2 |
| 13 | 2 |

Table S1 Legened. For each of the 12426 re-annotations, the number of bases by which the previously-annotated tail was extended.
